# Systematic review of guidelines for the diagnosis and treatment of Clostridioides difficile infection

**DOI:** 10.3389/fcimb.2022.926482

**Published:** 2022-08-30

**Authors:** Ting Gu, Wen Li, Li-Li Yang, Si-Min Yang, Qian He, Hai-Yu He, Da-Li Sun

**Affiliations:** ^1^ Department of Gastrointestinal Surgery, Second Affiliated Hospital of Kunming Medical University/Second Faculty of Clinical Medicine, Kunming Medical University, Kunming, China; ^2^ Department of Gastroenterology, Second Affiliated Hospital of Kunming Medical University/Second Faculty of Clinical Medicine, Kunming Medical University, Kunming, China

**Keywords:** *clostridioides difficile* infection, CDI, diagnosis, treatment, guidelines

## Abstract

**Objective:**

To systematically assess the current related methodological quality of guidelines for the diagnosis and treatment of Clostridioides difficile infection (CDI), revealing the heterogeneity and reasons for guideline recommendations for the diagnosis and treatment of CDI.

**Methods:**

We searched electronic databases systematically between 2017 and 2021 to find the latest guidelines for the diagnosis and treatment of CDI. The Appraisal of Guidelines for Research and Evaluation (AGREE II) tool was used for quality assessment of the included guidelines. The main recommendations for the diagnosis and treatment of CDI in the guidelines were extracted and evaluated for consistency, and the level of evidence supporting these recommendations was further extracted and analysed.

**Results:**

Fourteen guidelines for the diagnosis and treatment of CDI were finally included in this study. There were four guidelines, BSG and HIS, ASCRS, IDSA AND SHEA, and NICE, with an overall score of more than 60%, which is worthy of clinical recommendation. Further analysis of the consistency of the main recommendations for the diagnosis and treatment of CDI in the guidelines showed that the recommendations differed among guidelines. There are no unified classification criteria for the severity of CDI in current studies; some recommendations for the diagnosis and treatment of CDI do not provide evidence to support the recommendations, most recommendations cite low levels of evidence, and there is a lack of high-quality research evidence.

**Conclusion:**

The quality of guidelines for the diagnosis and treatment of CDI is uneven. Recommendations on the diagnosis and treatment of CDI have also varied in the guidelines for the past 5 years. Improvements of the aforementioned factors associated with causing heterogeneity would be a rational approach by guideline developers to further update guidelines for the diagnosis and treatment of CDI.

## Introduction

*Clostridioides difficile* (*C. difficile*) is a gram-positive, spore-forming anaerobic bacillus that is widely distributed in the gut and environment of humans and animals. Over the last decade, the frequency and severity of Clostridioides difficile infections (CDI) have been increasing worldwide and have become one of the most common hospital-acquired infections. Clinical manifestations are diverse, ranging from asymptomatic carrier status to varying degrees of diarrhoea to the most severe life-threatening colitis (Czepiel et al., 2019). Advanced age, antibiotic use, gastric acid suppression, recent hospitalisation or stay at nursing home, immunosuppression (HIV, cancer, organ transplantation) and infection with highly toxic strains are currently the main risk factors for CDI. A variety of treatment modalities are recommended, including vancomycin, fidaxomicin, and faecal microbiota transplantation (FMT) (Song and Kim, 2019). The diagnosis and treatment of CDI is challenging because of the general increase in drug resistance and there is no optimal laboratory test. Fortunately, the situation has been highly prioritized by researchers around the world. In recent years, many guidelines on how to diagnose and treat CDI have been developed (Crobach et al., 2018; Mullish et al., 2018; Abreu et al., 2019; Mullane et al., 2019; Sartelli et al., 2019; Antonelli et al., 2020; Gnocchi et al., 2020; Piekarska et al., 2020; Xu et al., 2020; Baunwall et al., 2021; Johnson et al., 2021; Kelly et al., 2021; Lewis et al., 2021; Poylin et al., 2021); however, the methodological quality of the guidelines is unclear, and they also vary widely regarding the items of diagnosis and treatment of CDI, which leads to confusion among guideline users. Therefore, the goal of this study is to analyse the methodological quality of current guidelines for the diagnosis and treatment of CDI using the AGREE II tool, reveal the heterogeneity of each guideline in the recommendations for the diagnosis and treatment of CDI, discuss the potential causes of this heterogeneity, help researchers and clinicians select the most appropriate guidelines and recommendations, and provide a better reference basis for guideline developers.

## Method

### Study design

In this study, the AGREE II tool was used to comprehensively evaluate and analyse the guidelines for the diagnosis and treatment of CDI, and the operation followed preferred reporting items for systematic review and meta-analysis protocols (PRISMA-P) (Shamseer et al., 2015).

### Search strategy

We searched electronic databases, namely, PubMed, Web of Science, Ovid, ScienceDirect, China Knowledge Network, and Wanfang Data. Given that the evidence is more updated and the results vary greatly when the time span is relatively large, we searched the guidelines for the diagnosis and treatment of CDI in the past 5 years to analyse and compare the evidence of the main recommendations. We searched the relevant guidelines for the diagnosis and treatment of CDI published between 2017 and 2021, and to obtain as many guidelines as possible, we also searched the public search tools Google and Baidu. The search strategy included “Clostridium difficile infection”, “diagnosis”, and “therapy”, in combination with the search terms “guideline”, “practice guideline”, “statement”, “recommendations”, and “consensus”. No language restrictions. The references of the included guidelines were also manually searched.

### Selection of guidelines

Inclusion criteria: (1) The target group included patients with CDI; (2) The guidelines focused on the diagnosis and treatment of CDI; (3) The full text was available online or in print; (4) The guidelines included both English and Chinese versions; (5) If the guidelines had an updated version, we used the latest version. Exclusion criteria: (1) repeated guidelines; (2) evaluation of the guidelines; (3) brief summary of the guidelines; (4) obsolete guideline version; and (5) narrative review. >Two independent reviewers conducted a systematic search of relevant guidelines for the diagnosis and treatment of CDI, screened and identified guidelines that met the inclusion criteria according to the above criteria, and invited a third reviewer to discuss the decision if there was controversy.

### Quality evaluation and method of assessing the guidelines

The quality of the guidelines was assessed using the most recent version of the AGREE II instrument, and the AGREE II instrument is a validated assessment tool designed to provide a framework for the assessment and monitoring of clinical guidelines, which measure and quantify the quality of guidelines (Wei et al., 2013). The AGREE II instrument consists of 23 items in the following six domains: Domain 1: Scope and purpose, addressing the overall goals of the guideline, specific health problems, and the target population (Items 1 to 3). Area 2: Participants, it is of interest who develops the guidelines and reflects the views and choices of the target population (Items 4 to 6). Area 3: Rigor of formulation, involving the process of gathering and screening evidence, the method of formulating recommendations and the process of updating recommendations (Items 7 to 14). Area 4: Clarity of presentation, in relation to the language, structure and format of the guide (Items 15 to 17). Area 5: Applicability, which includes barriers and facilitators that may be encountered during implementation, strategies to improve comprehension, and resource issues involved in applying this guide (Items 18 to 21). Area 6: Editorial independence, which relates to the formulation of recommendations that do not excessively favour competing interests (Items 22 to 23). Method: The four reviewers were uniformly web-trained and proficient in the use of the AGREE II tool. Each domain was evaluated independently by four reviewers, each of which was scored on a 7-point scale: 1 for strongly disagree and 7 for strongly agree. When little or no relevant information was provided, a score of 1 was given. A score of 2 to 6 was given when a statement did not fully meet the criteria or only one of the criteria was considered. When the criterion was closer or more considered, the score was higher. A score of 7 was given when the statement met all criteria or when all criteria were adequately considered. All items with a score difference of 3 or more were discussed further. Finally, a reviewer summarized all scores for each item and calculated the score for each domain using the following formula: (score obtained – minimum possible score)/(maximum possible score – minimum possible score) *100%. After review of the 23 items and comprehensive assessment by the reviewers, the evaluated guidelines were classified into the following three categories according to the AGREE II score: recommended, recommended after modification, and not recommended. The AGREE II manual does not provide guidance on how to interpret scores. To promote consistency in the evaluation of existing guidelines by the AGREE II instrument and to give recommendations on the level of evidence in all included guidelines, we used the following approach: guidelines with an overall score > 60% were recommended, guidelines with an overall score of 30%–60% were recommended after modification, and guidelines with an overall score < 30% were not recommended.

### Heterogeneity assessment of diagnosis and treatment items in guidelines

Relevant guidelines were scored using the agreement measurement scale (MSRA) (Pentheroudakis et al., 2008). Recommendations for the diagnosis and treatment of CDI were extracted based on key items in guidelines with scores of more than 60%, and the highest level of evidence for these recommendations was further determined by searching the database and analysing the citations of the included guidelines. This evidence was regraded by using the Oxford Centre for Evidence-Based Medicine (OCEBM) grading system (Howick et al., 0000).

### Statistical analysis

The normalized scores for each domain were calculated using the method of descriptive statistical analysis and expressed as a percentage; the median and range for each domain are presented. We used two-way ANOVAs to calculate intraclass correlation coefficients (ICCs) to test whether the scores of the four assessors were consistent. ICCs between 0.01 and 0.20 indicate a small agreement; 0.21–0.40, fair; 0.41–0.60, moderate; 0.61–0.80, substantial; and 0.81–1.00, very good. P < 0.05 was considered statistically significant. IBM SPSS version 19.0 (SPSS Inc., Chicago, IL, USA) was used for statistical analysis.

## Results

### Characteristics of the included guidelines

**Figure 1:**
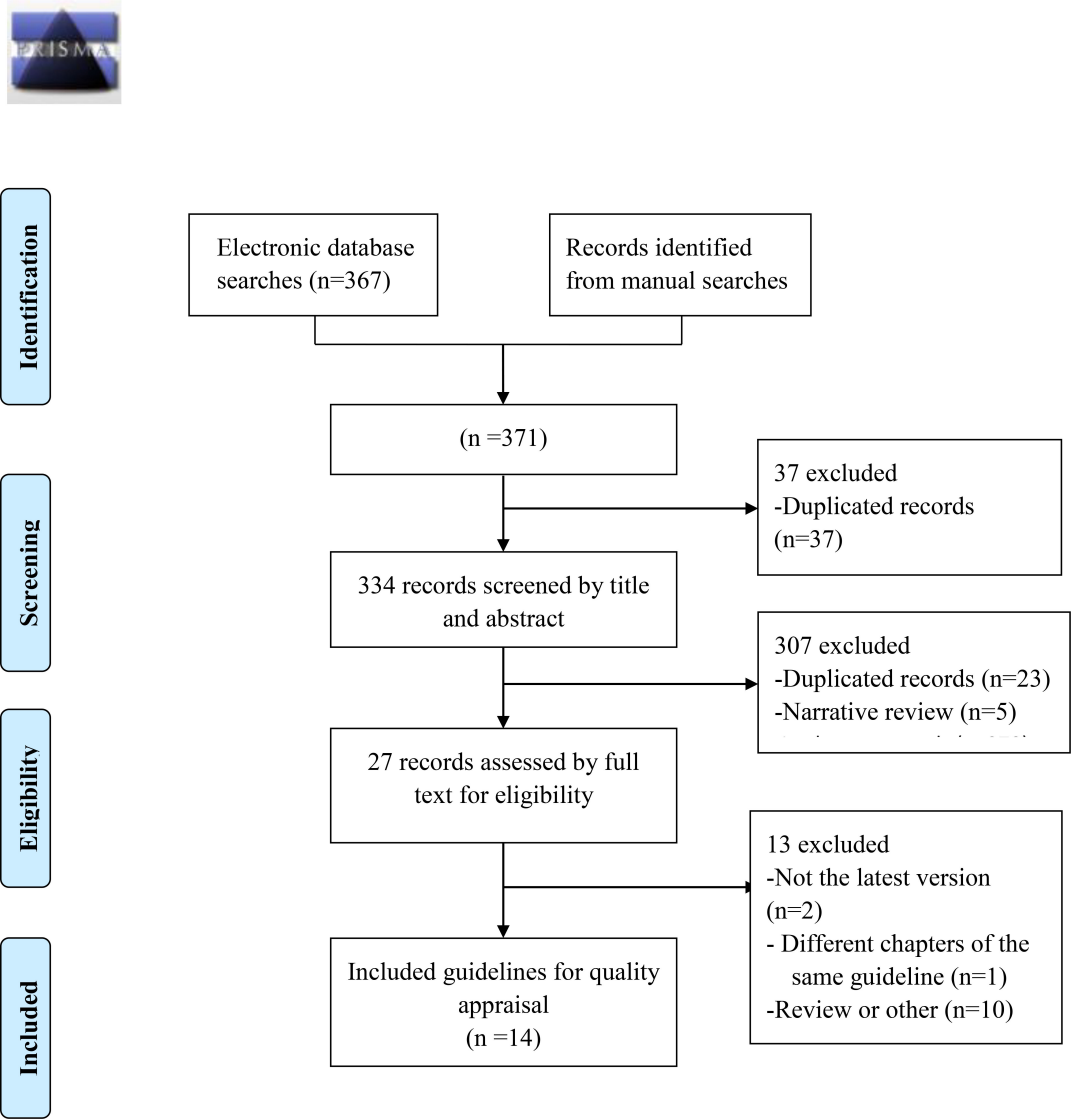
Study flow diagram.

A total of 371 articles were initially retrieved, and after screening by title, abstract and full text, fourteen guidelines on the diagnosis and treatment of CDI were included (**Figure 1**). The publication dates of the included guidelines ranged from 2017 to 2021. Seven of them used the GRADE grading system (Mullish et al., 2018; Abreu et al., 2019; Sartelli et al., 2019; Kelly et al., 2021; Poylin et al., 2021; Johnson et al., 2021; Lewis et al., 2021). One of them used the OCEBM grading system (Baunwall et al., 2021), and the other guidelines do not mention the grading of evidence (Crobach et al., 2018; Mullane et al., 2019; Antonelli et al., 2020; Gnocchi et al., 2020; Piekarska et al., 2020; Xu et al., 2020). One of the published guidelines was from Mexico (Abreu et al., 2019), one was from Germany (Antonelli et al., 2020), one was from the Netherlands (Crobach et al., 2018), one was from Italy (Gnocchi et al., 2020), one was from Poland (Piekarska et al., 2020), one was Danish (Baunwall et al., 2021), one was Chinese (Xu et al., 2020), one was developed by international organizations (Sartelli et al., 2019), two were British (Mullish et al., 2018; Lewis et al., 2021), and four were American (Mullane et al., 2019; Kelly et al., 2021; Poylin et al., 2021; Johnson et al., 2021). Eight of these guidelines were original versions (Crobach et al., 2018; Mullish et al., 2018; Abreu et al., 2019; Antonelli et al., 2020; Piekarska et al., 2020; Xu et al., 2020; Baunwall et al., 2021; Poylin et al., 2021), and six guidelines were updated from the original guidelines (Mullane et al., 2019; Sartelli et al., 2019; Gnocchi et al., 2020; Kelly et al., 2021; Johnson et al., 2021; Lewis et al., 2021). The eligible guideline characteristics are shown in **Table 1**.

**Table 1:** Characteristics of the included guidelines.

Guideline ID	Short name	Developmentorganization	Country applied	Grading system	Topic	Version	Target population	Development method
Abreu et al., 2019	AT	Mexico gastroenterological association	Mexico	GRADE	About clostridium refractory infection prevention, diagnosis and treatment of consensus	First	adult population	EB
Antonelli et al., 2020	MA	Springer-Verlag GmbH Germany	Germany	None	Clostridioides difficile (formerly Clostridium difficile) infection in the critically ill: an expert statement	First	critically ill patients	EB
Mullish et al., 2018	BH	BSG and HIS	British	GRADE	The use of faecal microbiota transplant as treatment for recurrent or refractory Clostridium difficile infection and other potential indications: joint British Society of Gastroenterology (BSG) and Health care Infection Society (HIS) guidelines	First	recurrent or refractory Clostridium difficile infection	EB
Sartelli et al., 2019	WS	WSES	Internal	GRADE	2019 update of the WSES guidelines for management of Clostridioides (Clostridium) difficile infection in surgical patients	Updated	surgical patients	EB
Crobach et al., 2018	MO	Centre for Infectious Diseases, Leiden University Medical Centre, Leiden, The Netherlands	Netherlands	None	Diagnostic Guidance for C. difficile Infections	First	Symptomatic or asymptomatic patients, and healthy persons	EB
Mullane et al., 2019	KA	American Society of Transplantation Infectious Diseases Community of Practice	American	None	Management of Clostridioides (formerly Clostridium) difficile infection (CDI) in solid organ transplant recipients: Guidelines from the American Society of Transplantation Community of Practice	Updated	Patients with solid organ transplants	EB
Gnocchi et al., 2020	MA	Department of Medicine and Surgery, University of Parma	Italy	None	Updated Management Guidelines for Clostridioides difficile in Paediatrics	Updated	Paediatrics	EB
Piekarska et al., 2020	AN	The polish society of epidemiology and infectious diseases	Poland	None	Clinical practice guidelines for Clostridioides(clostridium)difficile infection and faecal microbiota transplant protocol−recommendations of the polish society of epidemiology and infectious diseases	First	Patients infected with Clostridium difficile bacteria	EB
Xu YC et al., 2020	YI	The Chinese medical doctor association inspection physicians branch infectious disease medical expert committee	China	None	Chinese adult infection diagnosis and treatment of Clostridium difficile belongs expert consensus	First	Patients infected with Clostridium difficile bacteria	EB
Kelly et al., 2021	AC	ACG	American	GRADE	ACG Clinical Guidelines: Prevention, Diagnosis, and Treatment of Clostridioides difficile Infections	Updated	Patients infected with Clostridium difficile bacteria	EB
Poylin et al., 2021	AS	ASCRS	American	GRADE	The American Society of Colon and Rectal Surgeons Clinical Practice Guidelines for the Management of Clostridioides difficile Infection	First	Patients infected with Clostridium difficile bacteria	EB
Johnson et al., 2021	IS	IDSA AND SHEA	American	GRADE	Clinical Practice Guideline by the Infectious Diseases Society of America (IDSA) and Society for Health care Epidemiology of America (SHEA): 2021 Focused Update Guidelines on Management of Clostridioides difficile Infection in Adults	Updated	Adults infected with Clostridium difficile bacteria	EB
Baunwall et al., 2021	DS	DSGH	Danish	OCEBM	Danish national guideline for the treatment of Clostridioides difficile infection and use of faecal microbiota transplantation	First	Patients infected with Clostridium difficile bacteria	EB
Lewis et al., 2021	NI	NICE	British	GRADE	Clostridioides difficile infection: antimicrobial prescribing	Updated	Adults, young people and children infected with Clostridium difficile bacteria	EB

GRADE, The Grading of Recommendations Assessment, Development, and Evaluation; OCEBM, The Oxford Centre for Evidence-Based Medicine; BSG, British Society of Gastroenterology; HIS: Health care Infection Society; WSES, The World Society of Emergency Surgery; ACG, American College of Gastroenterology; ASCRS, American Society of colon and rectal surgeons; IDSA, The Infectious Diseases Society of America; SHEA, Society for Health care Epidemiology of America; DSGH, Danish Society of Gastroenterology and Hepatology; NICE, National Institute for Health and Care Excellence.

### Quality assessment of guidelines

The results of the assessment of the quality of all included guidelines using the AGREE II tool are shown in **Table 2**. The included guidelines had a low application score, the medians was 34.2% (17.7-71.9%). The scope, purpose, and independence of the editors had a small score gap. The median was 71.7% (range 29.2–90.3%) and 64.2% (range 4.2–97.9%), respectively. The medians of participant was 39.8% (20.8-88.9%). The medians of the formulated rigor and clarity of expression were 48.1% (18.8–84.9%) and 83.9% (62.5%–93.1%), respectively. Finally, depending on the score, we devised an overall recommendation. **Table 2** lists the detailed overall assessment for each guideline. It is important to note that there are two guidelines with relative scores exceeding 40% in each domain, which were developed by ASCRS (Poylin et al., 2021) and NICE (Lewis et al., 2021). Four guidelines have overall evaluation scores greater than 60% (Mullish et al., 2018; Poylin et al., 2021; Johnson et al., 2021; Lewis et al., 2021), these guidelines are recommended, and nine guidelines have overall evaluation scores between 30 and 60% (Abreu et al., 2019; Mullane et al., 2019; Sartelli et al., 2019; Antonelli et al., 2020; Gnocchi et al., 2020; Piekarska et al., 2020; Xu et al., 2020; Baunwall et al., 2021; Kelly et al., 2021), which are in the recommended category but still need improvement. There is one guideline with a total score of less than 30% (Crobach et al., 2018), which is not recommended. Four assessors participated in the assessment of the relevant guidelines. In the present study, the ICCs evaluated by the four evaluators for AGREE II were all greater than 0.8, illustrating the high consistency of the raters’ internal item scores.

**Table 2:** AGREE Ⅱ domain score and ICC of the included guidelines.

Guideline	Scope and purpose	Stakeholderinvolvement	Rigourof development	Claritypresentation	Applicability	Editorialindependence	Overallassessment
AT (Abreu et al., 2019)	80.6%	29.2%	58.3%	88.9%	25.0%	97.9%	57.9%
MA (Antonelli et al., 2020)	79.2%	31.9%	35.9%	81.9%	26.0%	56.3%	46.6%
BH (Mullish et al., 2018)	90.3%	88.9%	74.5%	62.5%	30.2%	72.9%	65.5%
WS (Sartelli et al., 2019)	56.9%	33.3%	57.3%	80.6%	28.1%	95.8%	54.7%
MO (Crobach et al., 2018)	29.2%	22.2%	18.8%	62.5%	40.6%	6.3%	29.9%
KA (Mullane et al., 2019)	75.0%	26.4%	22.9%	90.3%	22.9%	37.5%	40.1%
MA (Gnocchi et al., 2020)	83.3%	33.3%	32.8%	81.9%	17.7%	93.8%	49.2%
AN (Piekarska et al., 2020)	33.3%	20.8%	19.8%	81.9%	39.6%	4.2%	32.4%
YI (Xu et al., 2020)	77.8%	20.8%	20.8%	87.5%	31.3%	6.3%	37.1%
AC (Kelly et al., 2021)	61.1%	33.3%	39.1%	91.7%	38.5%	89.6%	53.9%
AS (Poylin et al., 2021)	83.3%	56.9%	77.1%	91.7%	42.7%	97.9%	71.2%
IS (Johnson et al., 2021)	81.9%	56.9%	71.4%	91.7%	35.4%	60.4%	63.1%
DS (Baunwall et al., 2021)	83.3%	25.0%	59.9%	93.1%	29.2%	97.9%	59.7%
NI (Lewis et al., 2021)	88.9%	77.8%	84.9%	88.9%	71.9%	81.3%	81.3%
ICC	0.938	0.989	0.984	0.917	0.959	0.977	−
Median score(range)	71.7% (29.2∼ 90.3%)	39.8% (20.8∼ 88.9%)	48.1% (18.8∼ 84.9%)	83.9% (62.5∼ 93.1%)	34.2% (17.7∼ 71.9%)	64.2% (4.2∼ 97.9%)	−

### Key recommendations and best evidence in guidelines for the diagnosis and treatment of CDI

**Figure 2:**
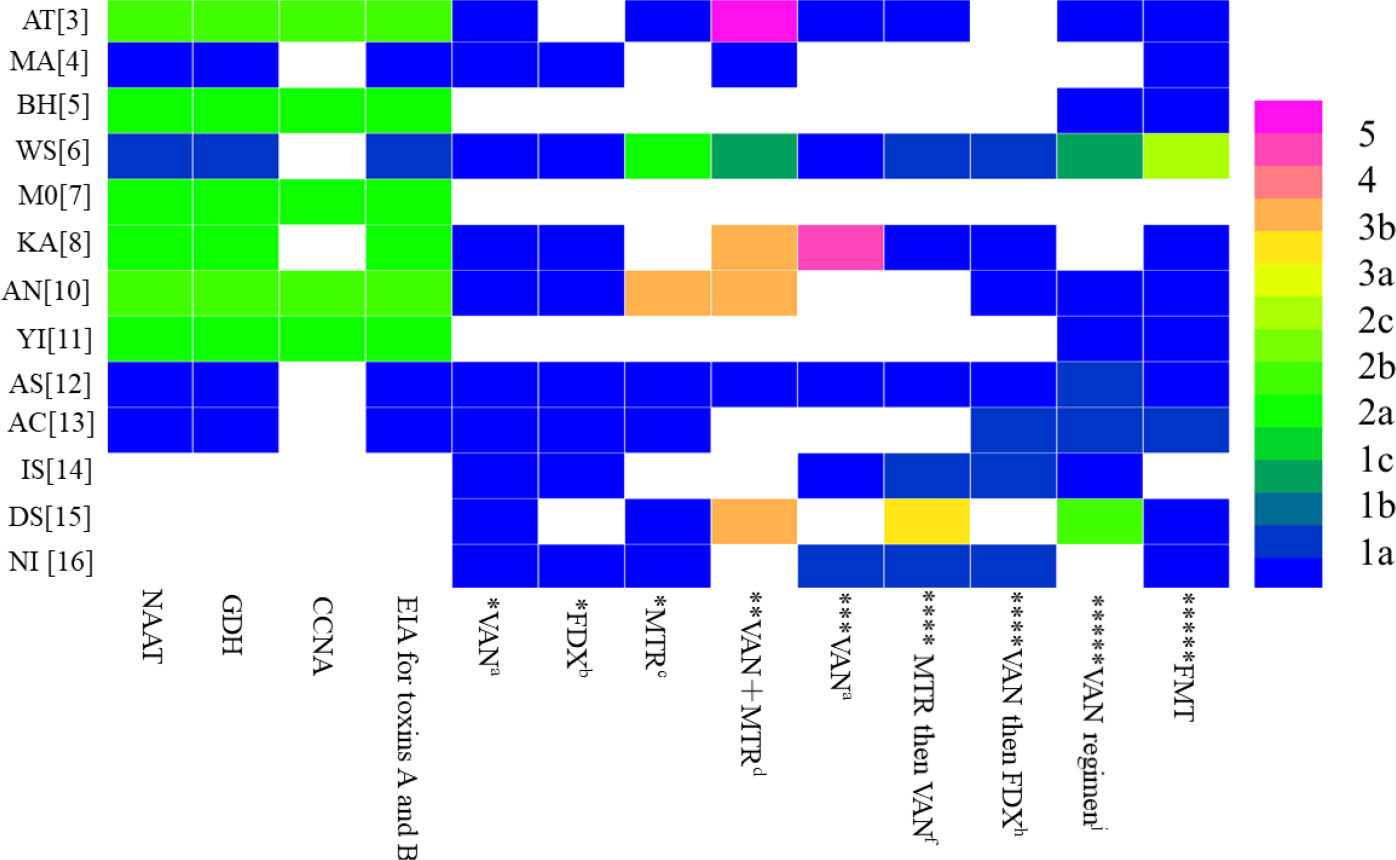
Distribution of the highest level of evidence to support similar recommendations for the diagnosis and therapy of Clostridioides difficile infection among the included guidelines. * represents the patient's first infection with C. difficile and is not serious; ** represents the patient's first infection with C. difficile and is Fulminant; *** represents thepatient's first infection with C. difficile and is serious; **** represents the patient's first recurrence of C. difficile infection; ***** represents the patient's multiple recurrent ofC. difficile infection.

To further analyse the reasons for the heterogeneity of recommendations for the diagnosis and treatment of CDI among different guidelines, we referred to the key recommendation items for the diagnosis and treatment of CDI in the high-quality guidelines (Kelly et al., 2021) and referred to these items to extract the key recommendations from among the fourteen included guidelines (**Table 3**). To further analyse the differences in key recommendations between different guidelines, we used MSRA; then, with the guidelines of the American College of Gastroenterology (ACG) (Kelly et al., 2021) for reference, the similarity of key recommendations was compared (**Table 4**), and OCEBM was used for regrading (**Figure 2**) to determine the effect of evidence selection on the strength of recommendations. Since the target population of the guideline is children, no further analysis was performed (Gnocchi et al., 2020) (**Figure 2**).

**Table 3:** Recommendations for the diagnosis and therapy of Clostridium difficile infection in the included guidelines.

GuidelineItem	AT[3]	MA[4]	BH[5]	WS[6]	MO[7]	KA[8]	AN[10]	YI[11]	AC [12]	AS [13]	IS [14]	DS[15]	NI[16]
**diagnosis**	
NAAT	●	●	●	●	●	⊕	●	●	●	●	—	⊕	—
2) TC	●	⊕	—	—	●	⊕	—	●	●	—	—	⊕	—
3) GDH	●	●	●	●	●	⊕	●	●	●	●	—	—	—
4) CCNA	●	—	●	—	●	—	●	●	●	—	—	—	—
5) EIA for toxins A and B	●	●	●	●	●	⊕	●	●	●	●	—	—	—
6) Flexible sigmoidoscopy	○	⊕	**—**	●	○	—	—	—	—	○	—	—	—
7) CCD	●	⊕	—	●	●	—	●	●	—	⊕	—	—	—
**treatment**	
*Initial episode, nonsevere													
VAN^a^	●	●	—	●	—	●	●	—	●	●	●	●	●
2) FDX^b^	—	●	—	●	—	●	●	—	●	●	●	—	●
3) MTR^c^	●	○	—	●	—	—	—	●	●	○	●	●	○
4) Probiotics+VAN	—	—	—	●	—	—	—	—	—	—	—	—	○
**Initial episode, Fulminant													
1) VAN^a^	—	—	—	—	—	●	—	—	●	—	●	●	●
2) total colectomy	—	—	—	●	—	—	●	—	—	●	—	●	—
3) VAN+MTR^d^	—	—	—	●	—	●	●	—	●	●	●	●	●
***Initial episode, Severe													
1) VAN^a^	●	—	—	●	—	●	●	●	●	—	●	●	●
2) FDX^b^	—	⊕	—	●	—	●	●	—	●	—	●	●	●
3) VAN+MTR^d^	●	●	—	—	—	●	—	●	—	●	—	—	—
4) Bezlotoxumab^e^	—	—	—	●	—	●	—	—	—	●	—	—	○
****First recurrence													
1) MTR then VAN^f^	●	—	—	●	—	●	●	—	●	●	●	●	●
2) VAN regimen^j^	●	—	—	—	—	—	—	●	●	●	●	—	○
3) VAN then FDX^h^	—	—	—	●	—	●	●	—	●	●	●	●	●
4) Bezlotoxumab^e^	—	—	—	—	—	—	—	—	—	●	●	—	○
*****Second or subsequent recurrence													
1) VAN regimen^j^	●	—	●	●	—	—	●	●	●	●	●	●	○
2) VAN then rifaximin^i^	—	—	—	—	—	●	—	—	—	●	●	—	⊕
3) FDX^b^	⊕	—	●	●	—	●	●	—	—	●	●	—	●
4) FMT	●	●	●	●	—	●	●	●	●	●	●	●	●
5) VAN+MTR^d^	●	—	—	—	—	—	—	—	—	●	—	—	—
6) Bezlotoxumab^e^	—	—	—	—	—	●	—	—	●	●	●	⊕	○

●: Indicates being recommended definitely; ⊕: indicates being mentioned; ○: indicates being not recommended; —: dedicates being not mentioned.

NAAT, nucleic acid amplification testing; TC, toxigenic culture; GDH, glutamate dehydrogenase; CCNA, cell cytotoxicity neutralization assay; EIA for toxins A and B, Toxin A and B enzyme immunoassays; CCD, Culture of Clostridium difficile; VAN, vancomycin; FDX, fidaxomicin; MTR, metronidazole; CDI,Clostridium difficile infection; FMT,faecal microbiota transplantation.

a: vancomycin 125 mg given 4 times daily for 10 days.

b: fidaxomicin 200 mg twice daily for 10 days.

c: When fidaxomicin and vancomycin are limited, metronidazole 500 mg 3 times per day by mouth for 10 days.

d: treatment with the combination of oral vancomycin (500 mg, 6 hourly) and intravenous metronidazole (500 mg, 8 hourly).

e: Bezlotoxumab 10 mg/kg infusion as an adjunct treatment.

f: vancomycin 125 mg given 4 times daily for 10 days if metronidazole was used for the initial episode.

j: vancomycin in a tapered and pulsed regime.

h: fidaxomicin 200 mg given twice daily for 10 days if vancomycin was used for the initial episode.

i: vancomycin, 125 mg 4 times per day by mouth for 10 days followed by rifaximin 400 mg 3 times daily for 20 days.

* represents the patient's first infection with C. difficile and is not serious; ** represents the patient's first infection with C. difficile and is Fulminant; *** represents the patient's first infection with C. difficile and is serious; **** represents the patient's first recurrence of C. difficile infection; ***** represents the patient's multiple recurrent ofC_2_ difficile infection.

**Table 4:** Scientific agreement of formulated recommendations for the diagnosis and therapy of Clostridium difficile infection in the included guidelines.

	AS[12]	AT[3]	MA[4]	BH[5]	WS[6]	M0[7]	KA[8]	AN[10]	YI[11]	AC[13]	IS[14]	DS[15]	NI[16]
NAAT	—	80-100%	80-100%	80-100%	80-100%	80-100%	80-100%	80-100%	80-100%	80-100%	—	80-100%	—
GDH	—	80-100%	80-100%	80-100%	80-100%	80-100%	80-100%	80-100%	80-100%	80-100%	—	—	—
CCNA	—	80-100%	—	80-100%	—	80-100%	—	80-100%	80-100%	—	—	—	—
EIA for toxins A and B	—	80-100%	80-100%	80-100%	80-100%	80-100%	80-100%	80-100%	80-100%	80-100%	—	—	—
*VAN^a^	—	80-100%	80-100%	—	80-100%	—	80-100%	80-100%	—	80-100%	80-100%	80-100%	80-100%
*FDX^b^	—	—	80-100%	—	80-100%	—	80-100%	80-100%	—	80-100%	80-100%	—	80-100%
*MTR^c^	—	80-100%	0-20 %	—	80-100%	—	—	—	80-100%	0-20 %	80-100%	80-100%	0-20 %
**VAN+MTR ^d^	—	—	—	—	80-100%	—	80-100%	80-100%	—	80-100%	80-100%	80-100%	80-100%
***VAN^a^	—	80-100%	—	—	80-100%	—	80-100%	80-100%	80-100%	—	80-100%	80-100%	80-100%
****MTR then VAN^f^	—	80-100%	—	—	80-100%	—	60-80 %	60-80 %	—	80-100%	60-80 %	60-80 %	60-80 %
****VAN then FDX^h^	—	—	—	—	60-80 %	—	60-80 %	60-80 %	—	80-100%	60-80 %	60-80 %	60-80 %
*****VAN regimen^j^	—	80-100%	—	80-100%	80-100%	—	—	60-80 %	80-100%	80-100%	80-100%	80-100%	0-20 %
*****FMT	—	80-100%	80-100%	80-100%	80-100%	—	80-100%	80-100%	80-100%	80-100%	80-100%	80-100%	80-100%

Measurement Scale of Rate of Agreement:

0%-20%: Radically different;

20%-40%: Numerous major scientific disagreements present;

40%-60%: Few major scientific disagreements present;

60%-80%: Only minor scientific disagreements present;

80%-100%: Absolute scientific agreement. In blank fields, no information is available.

NAAT, nucleic acid amplification testing; TC, toxigenic culture; GDH, glutamate dehydrogenase; CCNA, cell cytotoxicity neutralization assay; EIA for toxins A and B, Toxin A and B enzyme immunoassays; CCD, Culture of Clostridium difficile; VAN,vancomycin; FDX, fidaxomicin; MTR, metronidazole; CDI, Clostridium difficile infection; FMT, faecal microbiota transplantation.

a:vancomycin 125 mg given 4 times daily for 10 days.

b:fidaxomicin 200 mg twice daily for 10 days.

c:when fidaxomicin and vancomycin are limited, metronidazole 500 mg 3 times per day by mouth for 10 days.

d:treatment with the combination of oral vancomycin (500 mg, 6 hourly) and intravenous metronidazole (500 mg, 8 hourly).

e: bezlotoxumab 10 mg/kg infusion as an adjunct treatment.

f:vancomycin 125 mg given 4 times daily for 10 days if metronidazole was used for the initial episode.

j:vancomycin in a tapered and pulsed regime.

h:fidaxomicin 200 mg given twice daily for 10 days if vancomycin was used for the initial episode.

i:vancomycin, 125 mg 4 times per day by mouth for 10 days followed by rifaximin 400 mg 3 times daily for 20 days.

* represents the patient's first infection with C. difficile and is not serious; ** represents the patient's first infection with C. difficile and is Fulminant; *** represents the patient's first infection with C. difficile and is serious; **** represents the patient's first recurrence of C. difficile infection; ***** represents the patient's multiple recurrent of C. difficile infection.

The main recommendations and supporting evidence for the diagnosis and treatment of CDI are detailed in **Tables 2** and **3**. In terms of diagnosis, there were eleven references to NAAT (Crobach et al., 2018; Mullish et al., 2018; Abreu et al., 2019; Mullane et al., 2019; Sartelli et al., 2019; Antonelli et al., 2020; Piekarska et al., 2020; Xu et al., 2020; Baunwall et al., 2021; Kelly et al., 2021; Poylin et al., 2021), with ten mentions of GDH (Crobach et al., 2018; Mullish et al., 2018; Abreu et al., 2019; Mullane et al., 2019; Sartelli et al., 2019; Antonelli et al., 2020; Piekarska et al., 2020; Xu et al., 2020; Kelly et al., 2021; Poylin et al., 2021); there were six references to CCNA (Crobach et al., 2018; Mullish et al., 2018; Abreu et al., 2019; Piekarska et al., 2020; Xu et al., 2020; Kelly et al., 2021), with eleven mentions of EIA for toxins A and B (Crobach et al., 2018; Mullish et al., 2018; Abreu et al., 2019; Mullane et al., 2019; Sartelli et al., 2019; Antonelli et al., 2020; Piekarska et al., 2020; Xu et al., 2020; Kelly et al., 2021; Poylin et al., 2021)]. The coincidence rates of NAAT in the guidelines were 80-100% (Crobach et al., 2018; Mullish et al., 2018; Abreu et al., 2019; Mullane et al., 2019; Sartelli et al., 2019; Antonelli et al., 2020; Piekarska et al., 2020; Xu et al., 2020; Kelly et al., 2021; Baunwall et al., 2021; Poylin et al., 2021) (**Table 4**), and the highest level of evidence supporting this recommendation was 1a (**Figure 2**). The coincidence rates regarding GDH in the guidelines were 80–100% (Crobach et al., 2018; Mullish et al., 2018; Abreu et al., 2019; Mullane et al., 2019; Sartelli et al., 2019; Antonelli et al., 2020; Piekarska et al., 2020; Xu et al., 2020; Kelly et al., 2021; Poylin et al., 2021) (**Table 4**), and the highest level of evidence supporting this recommendation was 1a (**Figure 2**). The coincidence rates of CCNA in the guidelines were 80-100% (Mullish et al., 2018; Crobach et al., 2018; Abreu et al., 2019; Piekarska et al., 2020; Xu et al., 2020; Kelly et al., 2021) (**Table 4**), and the highest level of evidence supporting this recommendation was 2a (**Figure 2**). The coincidence rates of EIA for toxins A and B were 80–100% (Crobach et al., 2018; Mullish et al., 2018; Abreu et al., 2019; Mullane et al., 2019; Sartelli et al., 2019; Antonelli et al., 2020; Piekarska et al., 2020; Xu et al., 2020; Kelly et al., 2021; Poylin et al., 2021) (**Table 4**). The highest level of evidence supporting this recommendation was 1a (**Figure 2**). For the treatment of first-episode mild CDI, ten guidelines mentioned vancomycin, eight guidelines mentioned fidaxomicin, and nine mentioned metronidazole. The agreement rate regarding the use of vancomycin in all ten guidelines was 80–100% (**Table 4**), and the highest level of evidence supporting this recommendation was 1a (**Figure 2**). Eight guidelines reported agreement rates for fidaxomicin of 80–100%, and the highest level of evidence supporting this recommendation was 1a (**Figure 2**). Five of the nine guidelines had an 80-100% compliance rate for metronidazole (Abreu et al., 2019; Sartelli et al., 2019; Xu et al., 2020; Baunwall et al., 2021; Johnson et al., 2021), and three had a coincidence rate of 0%–20% (Antonelli et al., 2020; Lewis et al., 2021; Poylin et al., 2021). The highest level of evidence supporting this recommendation is 1a (**Figure 2**). For the treatment of first-episode severe CDI, vancomycin was mentioned in nine guidelines, with a coincidence rate of 80–100%, and the highest level of evidence supporting this recommendation was 1a (**Figure 2**). For the treatment of first-episode fulminant CDI, eight guidelines mentioned oral vancomycin combined with intravenous metronidazole, with a coincidence rate of 80–100%, and the highest level of evidence supporting this recommendation was 1a (**Figure 2**). For patients with a first relapse of CDI, nine guidelines mentioned the use of vancomycin at relapse if metronidazole was used at the first episode, and eight guidelines mentioned the use of fidaxomicin at relapse if vancomycin was used for the first episode. Three of the nine guidelines recommending the use of vancomycin for recurrence had a compliance rate of 80-100% (Abreu et al., 2019; Sartelli et al., 2019; Poylin et al., 2021), and five guidelines had a compliance rate of 60-80% (Mullane et al., 2019; Piekarska et al., 2020; Johnson et al., 2021; Baunwall et al., 2021; Lewis et al., 2021). The highest level of evidence supporting this recommendation was 1a (**Figure 2**). One of the eight guidelines recommending the use of fidaxomicin for recurrence had a compliance rate of 80-100% (Xu et al., 2020), and six guidelines had a compliance rate of 60-80% (Sartelli et al., 2019; Mullane et al., 2019; Piekarska et al., 2020; Johnson et al., 2021; Baunwall et al., 2021; Lewis et al., 2021). The highest level of evidence supporting this recommendation was 1a (**Figure 2**). For patients with multiple relapses of CDI, ten guidelines mentioned vancomycin dose-reduction therapy, and twelve guidelines mentioned faecal transplantation. Seven of the ten recommendations for vancomycin dose reduction therapy had a compliance rate of 80-100% (Mullish et al., 2018; Abreu et al., 2019; Sartelli et al., 2019; Xu et al., 2020; Baunwall et al., 2021; Johnson et al., 2021; Poylin et al., 2021), one guideline had a compliance rate of 60-80% (Piekarska et al., 2020), one guideline had a compliance rate of 0-20% (Lewis et al., 2021), and the highest level of evidence supporting this recommendation was 1a (**Figure 2**). The coincidence rate was 80-100% in all eleven guidelines using fidaxomicin, and the highest level of evidence supporting this recommendation was 1a (**Figure 2**).

## Discussion

### Principal findings

In the present study, we assess guidelines related to the diagnosis and treatment of CDI using the AGREE II tool. We discover that the quality of guidelines for the diagnosis and treatment of CDI varies greatly. Most recommendations are highly similar between guidelines, and there are large difference between guidelines in whether colonoscopy is used to diagnose CDI and whether metronidazole is used to treat it. The main reasons for the large difference include the following: there is no uniform definition for CDI, the recommendations for the diagnosis and treatment of CDI involve large differences in items, some indicators (MTR) recommendations are inconsistent, some recommendations do not provide evidence to support the recommendations, some recommendations lack high-quality evidence support, and some guidelines cite long years of evidence. In addition, the quality of guidelines varies significantly from guideline to guideline, or even from domain to domain for the same guideline.

### Quality evaluation guidelines of AGREE II

In the AGREE II scoring system, the three domains of rigor, participant, and applicability are scored lower.

There are very low scores on the rigor domain in the included guidelines. The reasons for this finding include the fact that some guidelines do not involve a search for evidence using a systematic search method (Crobach et al., 2018; Gnocchi et al., 2020; Piekarska et al., 2020; Xu et al., 2020; Kelly et al., 2021); most guidelines lack clear inclusion and exclusion criteria of evidence (Crobach et al., 2018; Abreu et al., 2019; Mullane et al., 2019; Sartelli et al., 2019; Antonelli et al., 2020; Gnocchi et al., 2020; Piekarska et al., 2020; Xu et al., 2020; Baunwall et al., 2021; Kelly et al., 2021); and some guidelines do not describe the recommendation formation process (Crobach et al., 2018; Mullane et al., 2019; Gnocchi et al., 2020; Piekarska et al., 2020; Xu et al., 2020); some guidelines do not specify the controversial parts (Crobach et al., 2018; Mullane et al., 2019; Sartelli et al., 2019; Gnocchi et al., 2020; Piekarska et al., 2020; Xu et al., 2020; Baunwall et al., 2021; Kelly et al., 2021); some guidelines, when formulating the recommendations, do not clearly indicate the benefits, side effects and risks to health (Baunwall et al., 2021); some guidelines are not reviewed by external experts before publication (Crobach et al., 2018; Abreu et al., 2019; Mullane et al., 2019; Sartelli et al., 2019; Antonelli et al., 2020; Gnocchi et al., 2020; Piekarska et al., 2020; Xu et al., 2020; Kelly et al., 2021); and some guidelines do not provide the guideline update process (Crobach et al., 2018; Mullish et al., 2018; Abreu et al., 2019; Mullane et al., 2019; Antonelli et al., 2020; Gnocchi et al., 2020; Piekarska et al., 2020; Xu et al., 2020; Baunwall et al., 2021; Kelly et al., 2021).

In the present study, the median total score of the participants domain among the included guidelines is moderate. The reason for this low score may be that the majority of associations that develop guidelines ignore the opinions of patients and the public (Crobach et al., 2018; Abreu et al., 2019; Mullane et al., 2019; Sartelli et al., 2019; Antonelli et al., 2020; Gnocchi et al., 2020; Piekarska et al., 2020; Xu et al., 2020; Baunwall et al., 2021; Johnson et al., 2021; Kelly et al., 2021; Poylin et al., 2021), and some guidelines do not clearly indicate the applicability of the guidelines (Crobach et al., 2018; Mullish et al., 2018; Abreu et al., 2019; Mullane et al., 2019; Sartelli et al., 2019; Gnocchi et al., 2020; Piekarska et al., 2020; Xu et al., 2020; Kelly et al., 2021; Baunwall et al., 2021). The needs of the patient and the opinions of the guideline user are essential for the development of guidelines, and clarifying the applicable group of guidelines helps the reader select the appropriate guideline as needed.

The median score of the included guidelines in application is very low. The reasons included the following: some guidelines do not describe facilitating and hindering factors in the application process (Mullish et al., 2018; Abreu et al., 2019; Mullane et al., 2019; Sartelli et al., 2019; Antonelli et al., 2020; Gnocchi et al., 2020; Piekarska et al., 2020; Xu et al., 2020; Baunwall et al., 2021; Kelly et al., 2021; Lewis et al., 2021; Poylin et al., 2021), some guidelines do not provide recommendations and supporting tools for applying the recommendations to clinical practice (Mullish et al., 2018; Abreu et al., 2019; Mullane et al., 2019; Sartelli et al., 2019; Antonelli et al., 2020; Gnocchi et al., 2020; Piekarska et al., 2020; Xu et al., 2020; Baunwall et al., 2021; Johnson et al., 2021; Poylin et al., 2021), and some guidelines do not consider potential resource investment problems when applying the recommendations (Crobach et al., 2018; Mullish et al., 2018; Abreu et al., 2019; Mullane et al., 2019; Sartelli et al., 2019; Antonelli et al., 2020; Gnocchi et al., 2020; Xu et al., 2020; Baunwall et al., 2021; Johnson et al., 2021; Kelly et al., 2021). The guidelines are developed to provide uniform standards and facilitate medical workers around the world. However, the medical resources and economic status of patients vary greatly in various regions, so it is necessary to give different suggestions according to the medical conditions of different countries and regions and in combination with the specific circumstances of patients.

### Analysis of the reasons for heterogeneity of guideline recommendations and evidence related to the diagnosis and treatment of CDI

(1) The target population of guidelines are different, including paediatric population (Piekarska et al., 2020; Lewis et al., 2021), critically ill patients (Antonelli et al., 2020), patients with recurrent CDI (Mullish et al., 2018), surgical patients (Sartelli et al., 2019), organ transplant patients (Crobach et al., 2018) and adults (Abreu et al., 2019; Piekarska et al., 2020; Xu et al., 2020; Baunwall et al., 2021; Johnson et al., 2021; Kelly et al., 2021; Poylin et al., 2021). The guidelines use different evidence for different populations, which leads to differences in recommendations.

(2) TC and CCNA diagnosis methods lack sufficient attention. Many guidelines do not have recommendations for TC (Mullish et al., 2018; Sartelli et al., 2019; Piekarska et al., 2020; Johnson et al., 2021; Lewis et al., 2021; Poylin et al., 2021) and CCNA (Sartelli et al., 2019; Mullane et al., 2019; Antonelli et al., 2020; Baunwall et al., 2021; Johnson et al., 2021; Lewis et al., 2021; Poylin et al., 2021). Diagnostic tests for CDI include toxigenic cultures (TC), cell culture cytotoxicity neutralization tests (CCCNA), toxin A and toxin B enzyme immunoassays (EIAs), nucleic acid amplification tests (NAATs), and glutamate dehydrogenase (GDH) tests. At present, the CDI diagnosis method adopts combination detection or multistep algorithms. The multistep algorithm consists of a combination of tests, that is, the GDH test plus EIAs, NAATs plus EIAs, or the GDH test plus EIAs, judged by NAATs in predefined stool sample testing criteria. Two-step screening is performed using the GDH test or NAATs, followed by testing of positive samples with EIAs to confirm the presence of Clostridium toxin production. Most guidelines recommend a two-step method for diagnosing CDI.

(3) Some recommendations are contrary: The flexible sigmoidoscopy diagnosis, metronidazole treatment, and bezlotoxumab prevention recommendations are in disagreement between different guidelines; partial guidelines for the recommended use of flexible sigmoidoscopy for the diagnosis of CDI (Sartelli et al., 2019). A prospective study (Johal et al., 2004), find that flexible sigmoidoscopy is superior to the faecal Clostridioides difficile cytotoxin test in a group of patients with pseudomembranous colitis. Therefore, it is concluded that sigmoidoscopy should be considered in all hospitalized patients with diarrhoea if stool is negative for Clostridioides difficile cytotoxin and enteric pathogens. The guidelines do not recommend the use of flexible sigmoidoscopy for the diagnosis of CDI (Crobach et al., 2018; Abreu et al., 2019; Poylin et al., 2021), and studies (Ben-Horin et al., 2010) have shown that pseudomembranes, which are considered to be the pathognomonic factors of CDI, are found in only approximately 45% to 55% of laboratory-confirmed CDI cases; moreover, sigmoidoscopy provides little additional diagnostic or prognostic value, poses the risk of complications such as perforation, and has limited clinical application, and therefore, flexible sigmoidoscopy is not recommended for the diagnosis of CDI. Some guidelines recommend the use of metronidazole for the treatment of CDI (Abreu et al., 2019; Sartelli et al., 2019; Xu et al., 2020; Baunwall et al., 2021; Johnson et al., 2021; Kelly et al., 2021). Efficacy analysis of one study (Appaneal et al., 2019) show that metronidazole is not different from vancomycin in the risk of all-cause mortality or recurrence of CDI within 30 days, and the cost of metronidazole is lower than that of vancomycin; guidelines do not recommend the use of metronidazole for the treatment of CDI (Antonelli et al., 2020; Lewis et al., 2021; Poylin et al., 2021). A Cochrane review (Nelson et al., 2017) show that in terms of its cure rate, vancomycin (79%) is more effective than metronidazole (72%; relative risk (RR) 0.90; 95% CI (0.84–0.97)), and metronidazole had potential side effects and a higher recurrence rate.

Metronidazole and vancomycin are the primary treatment options for CDI, but increasing rates of antimicrobial resistance and severe, refractory disease have prompted the need for alternative agents. Tigecycline has previously demonstrated favorable *in vitro* activity against C. difficile isolates, but clinical data on its use in the treatment of CDI are severely lacking. Some guidelines mention the use of tigecycline for the treatment of CDI (Sartelli et al., 2019; Antonelli et al., 2020; Xu et al., 2020; Kelly et al., 2021; Poylin et al., 2021), guidelines do not mention the use of tigecycline for the treatment of CDI (Crobach et al., 2018; Mullish et al., 2018; Abreu et al., 2019; Mullane et al., 2019; Gnocchi et al., 2020; Piekarska et al., 2020; Baunwall et al., 2021; Johnson et al., 2021; Lewis et al., 2021). Despite the heterogeneity of the included studies and the small number of patients, the available evidence suggests that tigecycline might be considered as a potential therapeutic option for patients with CDIs, especially in severe cases (Britt et al., 2014; Kechagias et al., 2020). Randomized controlled studies are needed for further evaluation of the effectiveness of tigecycline in the treatment of patients with CDI.

Partial guidelines for the recommended use bezlotoxumab of for the prevention of CDI recurrence (Mullish et al., 2018; Mullane et al., 2019; Sartelli et al., 2019; Piekarska et al., 2020; Baunwall et al., 2021; Johnson et al., 2021; Kelly et al., 2021; Poylin et al., 2021), a randomized study (Wilcox et al., 2017) show that bezlotoxumab achieve a significant benefit over placebo in the treatment of recurrent CDI. The guidelines do not recommend the use bezlotoxumab of for the prevention of CDI recurrence as the guideline consider that it is not cost effective (Lewis et al., 2021). The remaining guidelines are not clearly stated (Crobach et al., 2018; Abreu et al., 2019; Antonelli et al., 2020; Gnocchi et al., 2020; Xu et al., 2020).

(4) There is no uniform classification standard for the severity of CDI: For the definition of severe CDI, most guidelines consider it severe if either a leukocyte count of > 15 × 10^3^/µ L or a serum creatinine level of < 1.5 mg/dl is met (Abreu et al., 2019; Gnocchi et al., 2020; Piekarska et al., 2020; Xu et al., 2020; Johnson et al., 2021; Kelly et al., 2021; Poylin et al., 2021); guideline also indicate that albumin levels of < 2.5 g/dL may also indicate severe infection (Xu et al., 2020), and for this indicator of protein, the guidelines indicate that albumin < 30 g/L may indicate severe disease (Mullane et al., 2019; Baunwall et al., 2021). The guidelines indicate that creatinine ≥ 1.5 times the premorbid level may indicate severe disease (Sartelli et al., 2019; Baunwall et al., 2021); a baseline ≥ 133 μM/L should also be considered (Sartelli et al., 2019), and serum creatinine levels may be increased dramatically by more than 50% above the baseline (Lewis et al., 2021). Regarding the indicator of white blood cells, the guidelines state that a leukocyte count of < 2 ×10^9^/L may indicate severe disease (Baunwall et al., 2021). According to the guidelines, temperatures higher than 38.5 degrees Celsius are likely to indicate severe disease (Sartelli et al., 2019; Mullane et al., 2019; Lewis et al., 2021), and unformed bowel movements numbering > 10 BM/d should also be considered (Mullane et al., 2019). Differences in the recommendations for diagnosis and treatment are due to differences in severity grading of CDI. In addition, the cost of drugs can also affect treatment strategies. The guidelines (Abreu et al., 2019; Sartelli et al., 2019; Kelly et al., 2021) suggest that metronidazole is cheaper than vancomycin and fidaxomicin, metronidazole may be considered in the absence of vancomycin and fidaxomicin. The review found that vancomycin to be overall more effective than metronidazole for achieving symptomatic cure (79% vs 72%) and fidaxomicin to be more effective than vancomycin (71% vs 61%), although vancomycin financial costs is less expensive, lower recurrence rates of fidaxomicin imply overall similar cost-effectiveness for both agents (Nelson et al., 2017). For lower-risk patients (younger outpatients with minimal comorbidities), particularly in cost-sensitive environments, metronidazole is an appropriate alternative (Kelly et al., 2021). The guidelines suggest that metronidazole was neither clinically nor cost-effectiveness compared with vancomycin (Lewis et al., 2021).

Our study results provide the following references for the development of relevant guidelines for the diagnosis and treatment of CDI in the future: the update process of the guidelines should be clarified; the opinions of stakeholders, including users and patients, should be considered in the development of the guidelines; the conflicts of interest of guideline developers should be considered, and it should be clearly pointed out whether such conflicts of interest will affect the results of the guidelines; the relevant guidelines should be collected by a systematic method, with strict inclusion and exclusion criteria; the guidelines can be developed by multinational negotiation considering the geographical differences of population; the relevant experts should conduct an external review before publication of the guidelines; the latest evidence should be used to support the recommendations; and it is recommended that international academic groups develop a globally unified classification standard for CDI.

### Strengths and limitations

There are certain strengths and limitations of our study. The advantages of this study are as follows: (1) we not only collected the English version of the guidelines but also included such guidelines in other languages; (2) we sorted out the important diagnosis and treatment opinions of CDI, compared the evidence sources of each guideline, and analysed the heterogeneity and causes of the main recommendations. The limitations of this study are as follows: (1) to analyse the evidence, considering the update of the evidence, this study only included guidelines from the past 5 years and did not clarify older guidelines; (2) the AGREE II instrument can only focus on the method of developing guidelines, rather than assessing the effect of the recommendations on patients’ clinical outcomes.

## Conclusion

This study found that the quality of guidelines related to the diagnosis and treatment of CDI varies greatly, mainly because there are no unified classification criteria for the severity of CDI, some guidelines do not provide a source of evidence for their recommendations, and strict inclusion and exclusion criteria are lacking. It is recommended that higher-level studies be conducted to obtain higher-quality evidence and to improve the use of guidelines related to the diagnosis and treatment of CDI by addressing the above issues.

## Data availability statement

The original contributions presented in the study are included in the article/supplementary material. Further inquiries can be directed to the corresponding authors.

## Author contributions

All the authors contributed in the preparation of this work. TG, WL, and D-LS were drafted and revised the article; TG, WL, H-YH, and D-LS were responsible for the theme, final editing, and preparation of the manuscript for submission; H-YH and D-LS critically revised the manuscript. All authors read and approved the final manuscript.

## Funding

This study was supported by Yunnan young academic and technical leaders reserve talent project (No. 202105AC160049 to D-LS) and Yunnan Province Joint Special Project of Science & Technology Department of Yunnan Province and Kunming Medical University (No. 2019FE001-233 to H-HY).

## Conflict of interest

The authors declare that the research was conducted in the absence of any commercial or financial relationships that could be construed as a potential conflict of interest.

## Publisher’s note

All claims expressed in this article are solely those of the authors and do not necessarily represent those of their affiliated organizations, or those of the publisher, the editors and the reviewers. Any product that may be evaluated in this article, or claim that may be made by its manufacturer, is not guaranteed or endorsed by the publisher.

## Abbreviations

AGREE II, The Appraisal of Guidelines for Research and Evaluation II; ICC, Intra-class correlation coefficient; CDI, Clostridioides difficile infection; C. difficile, Clostridioides difficile; FMT, Faecal microbiota transplantation; GRADE, The Grading of Recommendations Assessment, Development, and Evaluation; OCEBM, The Oxford Centre for Evidence-Based Medicine; BSG, British Society of Gastroenterology; HIS, Health care Infection Society; WSES, The World Society of Emergency Surgery; ACG, American College of Gastroenterology; ASCRS, American Society of colon and rectal surgeons; IDSA, The Infectious Diseases Society of America; SHEA, Society for Health care Epidemiology of America; DSGH, Danish Society of Gastroenterology and Hepatology; NICE, National Institute for Health and Care Excellence; NAAT, nucleic acid amplification testing; TC, toxigenic culture; GDH, glutamate dehydrogenase; CCNA, cell cytotoxicity neutralization assay; EIA for toxins A and B, Toxin A and B enzyme immunoassays; CCD, Culture of Clostridioides difficile; VAN, vancomycin; FDX, fidaxomicin; MTR, metronidazole.

## References

[B1] Abreu A. Velasco J. Zavala-Solares M. R. Remes-Troche J. M. Carmona-Sanchez R. I. Aldana-Ledesma J. M.. (2019). Consensus on the prevention, diagnosis, and treatment of clostridium difficile infection. Rev. Gastroenterol. Mexico 84, 204–219. doi: 10.1016/j.rgmxen.2018.12.002 30987771

[B2] Antonelli M. Martin-Loeches I. Dimopoulos G. Gasbarrini A. Vallecoccia M. S. (2020). Clostridioides difficile (formerly clostridium difficile) infection in the critically ill: an expert statement. Intensive Care Med. 46, 215–224. doi: 10.1007/s00134-019-05873-x 31938827

[B3] Appaneal H. J. CaffreY A. R. LaPlante K. L. (2019). What is the role for metronidazole in the treatment of clostridium difficile infection? results from a national cohort study of veterans with initial mild disease. Clin. Infect. Dis. 69, 1288–1295. doi: 10.1093/cid/ciy1077 30561531

[B4] Baunwall S. M. D. Dahlerup J. F. Engberg J. H. Erikstrup C. Helms M. Juel M. A.. (2021). Danish National guideline for the treatment of clostridioides difficile infection and use of faecal microbiota transplantation (FMT). Scand. J. Gastroenterol. 56, 1056–1077. doi: 10.1080/00365521.2021.1922749 34261379

[B5] Ben-Horin S. Margalit M. Bossuyt P. Maul J. Shapira Y. Bojic D.. (2010). Prevalence and clinical impact of endoscopic pseudomembranes in patients with inflammatory bowel disease and clostridium difficile infection. J. Crohns Colitis 4, 194–198. doi: 10.1016/j.crohns.2009.11.001 21122505

[B6] Britt N. S. Steed M. E. Potter EM and Clough L. A. (2014). Tigecycline for the treatment of severe and severe complicated clostridium difficile infection. Infect. Dis. Ther. 3, 321–331. doi: 10.1007/s40121-014-0050-x 25466443 PMC4269622

[B7] Crobach M. J. T. Baktash A. Duszenko N. Kuijper E. J. (2018). Diagnostic guidance for c. Diff. Infect. Adv. Exp. Med. Biol. 1050, 27–44. doi: 10.1007/978-3-319-72799-8_3 29383662

[B8] Czepiel J. Drozdz M. Pituch H. Kuijper E. J. Perucki W. Mielimonka A.. (2019). Clostridium difficile infection: review. Eur. J. Clin. Microbiol. Infect. Dis. 38, 1211–1221. doi: 10.1007/s10096-019-03539-6 30945014 PMC6570665

[B9] Gnocchi M. Gagliardi M. Gismondi P. Gaiani F. De’ Angelis G. L. Esposito S. (2020). Updated management guidelines for clostridioides difficile in paediatrics. Pathogens 9, 291. doi: 10.3390/pathogens9040291 PMC7238231 32316346

[B10] Howick J. Chalmers I. Glasziou P. Greenhalgh T. Heneghan C. Liberati A.. (2011) Explanation of the 2011 Oxford centre for evidence-based medicine (OCEBM) levels of evidence (Background document) (Oxford Centre for Evidence-Based Medicine). Available at: http://www.cebm.net/index.aspx?o=5653.

[B11] Johal S. S. Hammond J. Solomon K. James PD and Mahida Y. R. (2004). Clostridium difficile associated diarrhoea in hospitalised patients: onset in the community and hospital and role of flexible sigmoidoscopy. Gut 53, 673–677. doi: 10.1136/gut.2003.028803 15082585 PMC1774022

[B12] Johnson S. Lavergne V. Skinner A. M. Gonzales-Luna A. J. Garey K. W. Kelly C. P.. (2021). Clinical practice guideline by the infectious diseases society of america (idsa) and society for healthcare epidemiology of america (shea): 2021 focused update guidelines on management of clostridioides difficile infection in adults. Clin. Infect. Dis. 73, e1029–e1e44. doi: 10.1093/cid/ciab549 34164674

[B13] Kechagias K. S. Chorepsima S. Triarides NA and Falagas M. E. (2020). Tigecycline for the treatment of patients with clostridium difficile infection: an update of the clinical evidence. Eur. J. Clin. Microbiol. Infect. Dis. 39, 1053–1058. doi: 10.1007/s10096-019-03756-z 31927652

[B14] Kelly C. R. Fischer M. Allegretti J. R. LaPlante K. Stewart D. B. Limketkai B. N.. (2021). ACG clinical guidelines: prevention, diagnosis, and treatment of clostridioides difficile infections. Am. J. Gastroenterol. 116, 1124–1147. doi: 10.14309/ajg.0000000000001278 34003176

[B15] Lewis T. Dancer S. Hand K. Hay A. Hill-Smith I. Howard P.. (2021). Clostridioides difficile infection: antimicrobial prescribing. Natl. Inst. Health Care Excellence (NICE) 23. 1–40. Available at: www.nice.org.uk/guidance/ng199.

[B16] Mullane K. M. Dubberke E. R. Practice A. I. C. (2019). Management of clostridioides (formerly clostridium) difficile infection (CDI) in solid organ transplant recipients: Guidelines from the American society of transplantation community of practice. Clin. Transplant. 33, e13564. doi: 10.1111/ctr.13564 31002420

[B17] Mullish B. H. Quraishi M. N. Segal J. P. McCune V. L. Baxter M. Marsden G. L.. (2018). The use of faecal microbiota transplant as treatment for recurrent or refractory clostridium difficile infection and other potential indications: joint British society of gastroenterology (BSG) and healthcare infection society (HIS) guidelines. Gut 67, 1920–1941. doi: 10.1136/gutjnl-2018-316818 30154172

[B18] Nelson R. L. Suda K. J. Evans C. T. (2017). Antibiotic treatment for clostridium difficile-associated diarrhoea in adults. Cochrane Database Syst. Rev. 3, CD004610. doi: 10.1002/14651858.CD004610.pub5 28257555 PMC6464548

[B19] Pentheroudakis G. Stahel R. Hansen H. Pavlidis N. (2008). Heterogeneity in cancer guidelines: should we eradicate or tolerate? Ann. Oncol. 19, 2067–2078. doi: 10.1093/annonc/mdn418 18662954 PMC2733109

[B20] Piekarska A. Panasiuk A. Stępień P. M. (2020). Clinical practice guidelines for clostridioides (Clostridium) difficile infection and fecal microbiota transplant protocol - recommendations of the polish society od epidemiology and infectious diseases. Przegl Epidemiol. 74, 69–87. doi: 10.32394/pe.74.06 32500988

[B21] Poylin V. Hawkins A. T. Bhama A. R. Boutros M. Lightner A. L. Khanna S.. (2021). The american society of colon and rectal surgeons clinical practice guidelines for the management of clostridioides difficile infection. Dis. Colon Rectum 64, 650–668. doi: 10.1097/DCR.0000000000002047 33769319

[B22] Sartelli M. Di Bella S. McFarland L. V. Khanna S. Furuya-Kanamori L. Abuzeid N.. (2019). 2019 update of the WSES guidelines for management of clostridioides (Clostridium) difficile infection in surgical patients. World J. Emerg. Surg. 14, 8. doi: 10.1186/s13017-019-0228-3 30858872 PMC6394026

[B23] Shamseer L. Moher D. Clarke M. Ghersi D. Liberati A. Petticrew Met.. (2015). Preferred reporting items for systematic review and meta-analysis protocols (PRISMA-p) 2015: elaboration and explanation. BMJ 350, g7647. doi: 10.1136/bmj.g7647 25555855

[B24] Song J. H. Kim Y. S. (2019). Recurrent clostridium difficile infection: Risk factors, treatment, and prevention. Gut Liver 13, 16–24. doi: 10.5009/gnl18071 30400734 PMC6346998

[B25] Wei D. Wang C.Y., W. Xiao X.J. Chen Y.L. C., Yao L., y., Liang F.X.. (2013). An example of the guideline research and evaluation (AGREE II) tool. Chin. JEvid-based Pediatr. 8, 316–319. doi: 10.3969/j.iss.1673-5501.2013.04.017

[B26] Wilcox M. H. Gerding D. N. Poxton I. R. Kelly C. Nathan R. Birch T.. (2017). Bezlotoxumab for prevention of recurrent clostridium difficile infection. N Engl. J. Med. 376, 305–317. doi: 10.1056/NEJMoa1602615 28121498

[B27] Xu Y.C. Zhang M.. The Chinese medical doctor association inspection physicians branch infectious disease medical expert committee (2020). Chinese Adult infection diagnosis and treatment of clostridium difficile belongs expert consensus. Med. J. Peking Union Med. Coll. Hosp. 131-38. doi: 10.3969/j.issn.1674-9081.2017.03.010

